# A Cost Analysis of Mobile Integrated Health for Acute Care

**DOI:** 10.5811/westjem.48521

**Published:** 2026-02-12

**Authors:** Laurel O’Connor, Olivia Dunn, Sophia Merolle, Cosette Salaun, Bettina Valentiner, Joel Rowe, Alexander Ulintz, Timothy Boardman, Jan M Otero, Martin Reznek, Scott A Goldberg, Renata Konrad

**Affiliations:** * University of Massachusetts Chan Medical School, Department of Emergency Medicine, Worcester, Massachusetts; † Worcester Polytechnic Institute, School of Business, Worcester, Massachusetts; ‡ University of Florida, Department of Emergency Medicine, Gainesville, Florida; § The Ohio State University College of Medicine, Department of Emergency Medicine, Columbus, Ohio; || Fire Division, Lake County Board of County Commissioners, Mascotte, Florida; # Brigham and Women’s Hospital, Department of Emergency Medicine, Boston, Massachusetts

## Abstract

**Objectives:**

Mobile integrated health programs have emerged as a means to reduce avoidable emergency department (ED) visits and optimize healthcare resource utilization. Such models are estimated to cost less than ED encounters but may be more costly than traditional ambulatory services. However, mobile integrated health is not reimbursed by most payors, and its operational costs are poorly understood. Our objective if this study was to estimate the costs of delivering acute care services through a mobile integrated health program.

**Methods:**

This study was performed at an urban academic tertiary care center with a hospital-affiliated emergency medical services agency in which a mobile integrated health program is embedded. Home visits are conducted by paramedics who collaborate with a remotely located, actively engaged physician to evaluate and treat patients. We compiled cost data derived from real-world mobile integrated health patient encounters to account for all the resources needed to perform acute care visits. Mobile integrated health visits were categorized as basic, involving lower complexity evaluations with limited diagnostics, or advanced, which include higher acuity care with intravenous medications and multiple diagnostic studies. We used Monte Carlo simulations to provide probabilistic estimates of the cost per visit of mobile integrated health-facilitated care.

**Results:**

Using a Monte Carlo simulation with 1,000 iterations, we established cost estimates for basic and advanced service categories of mobile integrated health services. The median cost of a basic call is $550 (90% CI [$512–$676]), and $1400.00 for an advanced call (90% CI [$810–$1,813]).

**Conclusion:**

This project, which generated real-world cost estimates for mobile integrated health programs delivering acute care services, offers essential context for policymakers and payors evaluating sustainable reimbursement models. We estimate that mobile integrated health services cost more than the mean cost of most outpatient clinic visits ($160) but remain substantially less expensive than emergency department visits ($2,715) or inpatient admissions ($24,680). These findings should be interpreted with caution, given the limitations of simulation-based estimates in a single system. They highlight the ongoing need to prospectively and rigorously assess the cost-effectiveness of mobile integrated health models.

## INTRODUCTION

As health systems face increasing pressure to mitigate limited ambulatory access, hospital crowding, and rising costs, mobile integrated health programs have emerged as a promising model for delivering high-value, community-based care.^1^–^4^ Defined broadly as the use of traditional out-of-hospital personnel and resources in novel ways, typically in a non-transport capacity, mobile integrated health programs provide a coordinated spectrum of health services, spanning preventative, acute, and transitional care, directly in community settings.^2^, ^5^–^7^ They aim to reduce avoidable emergency department (ED) visits and hospitalizations by expanding access to timely, patient-centered care, particularly for high-risk populations with chronic or complex medical needs.^2^, ^5^–^7^ Mobile integrated health programs can improve clinical outcomes, enhance satisfaction among patients and clinicians, and consolidate acute-care resource use within healthcare systems.^8^–^10^

Despite growing evidence of clinical benefit, mobile integrated health programs face inconsistent reimbursement, which challenges their sustainability and scalability.^11^–^13^ Unlike hospital- and clinic-based care, mobile integrated health lacks standardized billing codes for services, forcing programs to rely on institutional or municipal support, grant funding, or individually negotiated contracts.^11^,^12^ Consequently, despite demonstrated clinical effectiveness, many of these programs fail due to financial instability or even fail to successfully launch.^14^ The economic value of mobile integrated health programs has not been rigorously described, making it difficult to advocate for policymakers to recognize the need for consistent payment structures.^11^ Mobile integrated health programs require specialized personnel, transportation, equipment, and logistical coordination, making them relatively resource-intensive compared to those required for ambulatory clinic-based care. However, they also have the potential to reduce the need for expensive brick-and-mortar acute care settings like EDs.^15^ To date, most economic analyses of mobile integrated health have been retrospective, observational studies that may not fully capture programs’ financial and operational complexities or directly measure them.^4^,^6^,^16^

Simulation modeling offers a powerful alternative, allowing researchers to evaluate the cost of mobile integrated health under various real-world scenarios.^17^,^18^ By modeling patient trajectories, resource utilization, and clinical outcomes, simulation-based analyses can provide actionable insights into financial sustainability and potential return on investment of mobile integrated health programs.^18^ Our objective in this study was to estimate the operational costs of an urban mobile integrated health program. By applying simulation-based methods, we sought to provide economic data to inform future funding models and help guide policy decisions on the role of mobile healthcare in health systems.

## METHODS

We performed this study at an urban, academic, tertiary-care medical center with a hospital-owned emergency medical services (EMS) agency and an embedded mobile integrated health program. The program of interest performs approximately 800 annual visits to 12 cities and towns in the service area, with six full-time community paramedics and four medical directors.^8^,^19^ In this model, mobile paramedics perform all home visits equipped with mobile diagnostics and a portable formulary, supported in real time by a remotely located on-call medical director through a secure telehealth platform. This cost evaluation is limited to the analysis of acute care services from 2022–2024. This project was deemed “not human subjects research” by the affiliated institutional review board.

**Population Health Research Capsule**

What do we already know about this issue?

*Mobile integrated health programs can reduce emergency department (ED) use but lack reimbursement frameworks and sufficiently characterized operational costs*.

What was the research question?

*What are the real-world costs of delivering acute mobile integrated health services?*

What was the major finding of the study?

*Using Monte Carlo simulation models, we found that mobile integrated health visits cost $512–$676 for basic care and $810–$1,813 for complex care*,.

How does this improve population health?

*By clarifying mobile integrated health costs, this study supports reimbursement models that expand access to home-based acute care and reduce costly ED use*.

We defined “basic” mobile integrated health visits as lower complexity evaluations requiring a single type of diagnostic test (laboratory or radiology study) and no intravenous (IV) medications, and “advanced” mobile integrated health visits as those involving two or more diagnostic modalities and/or IV therapies. These definitions were selected to align mobile integrated health visit complexity with established care bundles used in emergency department settings.^20^ Mobile integrated health visit volumes and the proportion of each visit complexity category were based on data from the study period. In practice, approximately 60% of encounters were classified as basic, consisting of a bedside paramedic evaluation, a telehealth physician consultation, and referral for appropriate follow-up. These visits often included one diagnostic study (laboratory or radiologic), administration of oral medications, or prescription of a new medication. The remaining 40% of visits were classified as advanced, encompassing all elements of basic care along with higher acuity interventions such as administering IV medications or fluids and both laboratory and radiologic testing.

We employed a cost-analysis Monte Carlo simulation to estimate the cost per mobile integrated health visit based on existing clinical and operational data. A priori power analysis was not performed because this was an economic modeling study rather than a hypothesis-driven investigation. To address the multifaceted variables that influence healthcare costs during discrete encounters, we employed repeated random simulations to estimate outcomes, exploring diverse variable combinations.^21^,^22^ Simulations were run 1,000 times to establish a 90% probabilistic confidence interval around cost estimates. Simulations were performed using Palisade @Risk (Lumivero, Denver, CO).^23^ We abstractred deidentified clinical data from the electronic health record using Web Intelligence v4 (SAP BusinessObjects, Walldorf, Germany) reporting. We abstracted operational data in aggregate from RescueNet (Zoll Medical Corporation, Chelmsford, MA). Administrative financial data (eg, staff salaries) were reported manually. Supplemental Table 1 summarizes the costing elements incorporated into the model. Supplemental Table 2 describes cost elements in detail.

Program operating costs were aggregated and classified as fixed or variable. To account for inherent variability in each cost estimate, we applied a program evaluation and review technique (PERT) probability distribution to both fixed and variable costs.^24^ Distributions using PERT are a validated approach in economic modeling, providing a natural representation of uncertainty by emphasizing the most likely estimate while allowing realistic variation. Compared to triangular distributions, PERT provides a more natural representation of uncertainty by reducing the influence of extreme values.^24^ The PERT distribution uses the minimum, most likely, and maximum values, producing a bell-shaped curve that places greater weight on the most probable estimate while allowing for variation within a defined realistic range. Table 1 characterizes each variable. We analyzed cost patterns for each category, including the number of visits, diagnostic tests, medications, and clinical supplies per visit.

Subsequently, we performed a structured cost allocation incorporating fixed and variable expenses. Fixed costs were calculated as a total sum, proportionally allocated based on the distribution of basic vs advanced visits. We calculated variable costs by multiplying the unit costs of diagnostic tests and medications by the volume of each visit type. The fixed and variable costs were summed and then divided by the number of visits to estimate the cost per visit for basic and advanced calls separately. As radiology studies and lab analyses are already reimbursed by payors, we elected to perform an additional cost analysis, excluding laboratory analysis and radiology interpretation, to model total unreimbursed mobile integrated health costs. The primary study outcomes were the estimated costs of basic and advanced mobile integrated health visits. This study was reported in accordance with the Consolidated Health Economic Evaluation Reporting Standards guidelines.^25^

## RESULTS

The estimated median total cost per basic MIH visit was $550 (90% CI [$512–$676]). For advanced calls, the estimated median total cost per visit was $1400 (90% CI [$810–$1,813]). When excluding lab and radiology costs, the estimated median unreimbursed cost per basic visit was $489 (90% CI [$461–$516]) and the estimated median unreimbursed cost per advanced visit was $557 (90% CI [$536–$591]). The figure summarizes the distribution of all costs associated with basic and advanced calls, demonstrating the probabilistic nature of the analysis.

## DISCUSSION

Establishing a standardized reimbursement structure is essential in assessing and advancing the sustainability of mobile integrated health programs.^11^–^13^ Many of these programs rely on institutional or municipal funding or grants, limiting scalability and sustainability. This study provides estimates of the cost per encounter for mobile integrated health when used to provide acute care. By analyzing the distribution of simulated outcomes, we were able to quantify uncertainty levels and identify the most influential factors affecting costs across various scenarios.

Compared with the mean cost of an outpatient clinic visit ($160), ED visit ($2,715), and inpatient admission ($24,680), mobile integrated health services are more costly than a typical outpatient encounter but substantially less expensive than ED visits and hospitalizations.^26^,^27^ Framing these operational expenditures in relation to potential downstream utilization highlights their role as inputs for formal cost-effectiveness analyses. Mobile integrated health should, therefore, be considered not only in terms of absolute operating costs but also in terms of the incremental value it provides relative to usual care. Our estimates offer foundational data for future cost-effectiveness modeling, which will be essential to inform payors and policymakers about the value proposition of mobile integrated health programs. Prospective cost-effectiveness evaluations are needed to validate these preliminary findings. Demonstrated downstream savings, such as avoided hospitalizations, reductions in iatrogenic complications, and mitigation of delays in care, may further accentuate the perceived cost advantages of mobile integrated health. Finally, we note that this pilot program was shaped by available funding and staffing. With greater investment, economies of scale may be achieved, further reducing per capita costs.

There are also non-economic advantages of mobile integrated health: Addressing healthcare needs at home may increase ED capacity and improve throughput, allowing ED and EMS assets to focus care on the highest acuity patients, thereby decreasing ED wait times and left-without-being-seen rates, and decreasing ED boarding, which are quality drivers within health systems.^28^–^31^ Finally, mobile integrated health provides dignified care at home in a way that aligns with patient preferences.^2^,^32^,^33^

Despite its benefits, the mobile integrated health delivery model requires specialized personnel, mobile infrastructure, and coordination with existing healthcare entities, which renders it resource-intensive. Operational leaders should target their resources toward patients most at risk of high-cost healthcare use patterns. This targeted approach is necessary to optimize resources and ensure that mobile integrated health models are acceptable to payors, who may be concerned about overutilization.^11^ As healthcare systems shift toward value-based care, mobile integrated health represents a strategic opportunity to align financial incentives with improved patient outcomes, particularly for high-risk populations with chronic complex conditions, if it can be shown to be cost effective.^11^

## LIMITATIONS

This study had limitations. The analysis considers a single mobile integrated health model focusing on acute care, limiting its generalizability. The structure and resources of the program may differ from those of other services. Overhead and training costs, including taxes, utilities, and vehicle purchases, were not included in estimates. This project was limited to cost estimation; cost-effectiveness analysis, an important measure of program sustainability, was beyond the study’s scope. The findings presented rely on simulated data and not a prospective evaluation of real mobile integrated health visits. We did not perform additional one-way or multi-way sensitivity analyses; however, the use of Monte Carlo simulation with PERT distributions provided a probabilistic sensitivity analysis that partially addresses robustness. This study reports per-visit costs rather than total annual or per-capita program costs; while the methodology could be extended to produce such estimates, this was outside the scope of the present analysis.

This study was based on a single, urban, academic mobile integrated health program and may not be generalizable to other health systems, geographic regions, or organizational models. While our uncertainty analysis used validated methods, simulation-based estimates rely on assumptions about input costs, visit complexity, and program operations, which may not fully reflect the heterogeneity of real-world mobile integrated health implementation. These assumptions, while grounded in observed program data, should be considered when interpreting the findings. Future research should include prospective cost-effectiveness analysis in real clinical practice using a validated costing approach, such as time-driven, task-based activity costing.^33^

Mobile integrated health may offer a solution for managing acute care in the community. As payors and health systems seek solutions to rising healthcare expenditures and capacity constraints, efforts to prospectively measure cost-effectiveness will help assess the value of mobile integrated health in balancing the competing priorities of access, quality, and cost in modern health care delivery.

## Supplementary Information





## Figures and Tables

**Figure:**
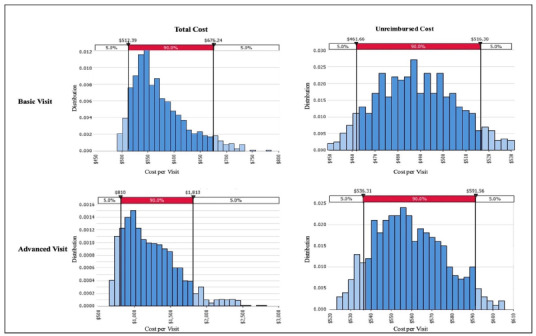
Estimated mobile integrated healthcare cost per visit using Monte Carlo simulations.

**Table 1:** Description of fixed and variable costs used to estimate the per-visit cost of a mobile integrated health program, Visits were categorized as “basic” or “advanced” based on the complexity of the resources needed to perform the visit.

Cost type	Cost category	Components
Fixed	Personnel salaries with fringe*	Paramedics (6.5 full-time effort) Program administrator (1 full-time equivalent) Physician medical direction (0.5 full-time equivalent)
	Administrative costs*	State license fees Paramedic certification fees Continuing education courses Operational software
	Non-disposal clinical equipment	Cardiac monitors Point-of-care blood machine Laptop computers Portable printers Radios WIFI Hotspot Clinical software
Variable	Total annual number of encounters* Fuel* Vehicle maintenance* Laboratory analysis* Radiology studies and interpretation* Visit complexity*	
	Basic (60% of visits)	Bedside paramedic evaluation Telehealth physician evaluation Referral for appropriate follow-up Plus, any of the following: One type of diagnostic study (lab tests or radiology) Administration of oral medications (one or more) Prescription for a medication that is not over the counter
	Advanced (40% of visits)	Basic services as above Plus, any of the following: One or more intravenous medications including fluids Two types of diagnostics studies (lab test and radiology study)

* Program evaluation and review technique was applied to account for variability and uncertainty in costs over time for fixed costs.
