# Supplementary material for: A Cost Analysis of Mobile Integrated Health for Acute Care
Source: West J Emerg Med. 2026 Feb 12;27(2):445–51. doi: 10.5811/westjem.48521 (PMC13016050; doi:10.5811/westjem.48521)
Supplement: Supplementary file 1 [file wjem-27-445-s001.docx]

| Supplementary Table 1: Summary of Cost Components: Summary of major cost components included in the MIH program costing analysis, showing representative ranges for expenditures. | |
| --- | --- |
| Category | Cost Range / Value |
| Nondisposable Clinical Equipment | $16,327 – $19,955 |
| Paramedic Salaries (6.5 FTE) | $82,000 – $106,000 each |
| Physician Salary Support | $110,000 – $160,000 |
| Administrator | $60,000 – $81,000 |
| Administrative Costs | $10,000 – $15,000 |
| Vehicle Maintenance | $4,082 – $4,989 |
| Fuel (per mile) | $2.15 – $3.61 |
| Disposable Clinical Equipment (per call) | $0 – $21.14 |
| Medications (per unit) | $0.90 – $558.93 |
| Diagnostic Test Analysis (per test) | $9.44 – $461.00 |
