# Supplementary material for: A Cost Analysis of Mobile Integrated Health for Acute Care
Source: West J Emerg Med. 2026 Feb 12;27(2):445–51. doi: 10.5811/westjem.48521 (PMC13016050; doi:10.5811/westjem.48521)
Supplement: Supplementary file 2 [file wjem-27-s002.docx]

| Supplemental Table 2: All Costing Elements with Ranges | | | | | | | |
| --- | --- | --- | --- | --- | --- | --- | --- |
| Visit Information | Volume | Number of MIH Visits | Mean | 850 |  |  |  |
|  |  |  | Minimum | 560 |  |  |  |
|  |  |  | Maximum | 1140 |  |  |  |
|  | Complexity | Proportion of Basic Visits | Mean | 60% | Proportion of Advanced Visits | Mean | 40% |
|  |  |  | Minimum | 54% |  | Minimum | 30% |
|  |  |  | Maximum | 66% |  | Maximum | 50% |
|  |  |  |  |  | Proportion of Advanced Visits Redirected to the ED | Mean | 6% |
| Cost Components | Fixed Costs | Nondisposable Clinical Equipment | Mean | 18,141.00 |  |  |  |
|  |  |  | Minimum | 16,326.90 |  |  |  |
|  |  |  | Maximum | 19,955.10 |  |  |  |
|  |  | Paramedic Salaries with Fringe | $82,000-$106,000 |  |  |  |  |
|  |  | Number of Paramedics FTE | 6.2 |  |  |  |  |
|  |  | Physician Salary Support | $110,000-160,000 |  |  |  |  |
|  |  | Administrator | $60,000-81,000 |  |  |  |  |
|  |  | Administrative Costs | $10,000-$15,000 |  |  |  |  |
|  | Variable Costs | Miles Traveled | Mean | 3102.00 |  |  |  |
|  |  |  | Minimum | 2791.80 |  |  |  |
|  |  |  | Maximum | 3412.20 |  |  |  |
|  |  | Cost of Fuel per mile | Mean | $2.88 |  |  |  |
|  |  |  | Minimum | $3.61 |  |  |  |
|  |  |  | Maximum | $2.15 |  |  |  |
|  |  | Vehicle Maintenance | Mean | $4,535.00 |  |  |  |
|  |  |  | Minimum | $4,081.50 |  |  |  |
|  |  |  | Maximum | $4,988.50 |  |  |  |
|  |  | Disposable Clinical Equipment (annually) | Mean | $4.75 |  |  |  |
|  |  |  | Minimum | $0.00 |  |  |  |
|  |  |  | Maximum | $21.14 |  |  |  |
|  |  | Medications (per unit) | Mean | $17.39 |  |  |  |
|  |  |  | Minimum | $0.90 |  |  |  |
|  |  |  | Maximum | $558.93 |  |  |  |
|  |  | Number of Medications Per Visit (basic) | Mean | 0.37 | Number of Medications Per Visit (advanced) | Mean | 1.4 |
|  |  |  | Minimum | 0.00 |  | Minimum | 1 |
|  |  |  | Maximum | 1.00 |  | Maximum | 4 |
|  |  | Diagnostic Test Analysis | Mean | $68.91 |  |  |  |
|  |  |  | Minimum | $9.44 |  |  |  |
|  |  |  | Maximum | $461.00 |  |  |  |
|  |  | Number of diagnostic tests performed (basic) | Mean | 0.41 | Number of laboratory tests performed (advanced) | Mean | 4.6 |
|  |  |  | Minimum | 0.00 |  | Minimum | 2 |
|  |  |  | Maximum | 1.00 |  | Maximum | 9 |
